# The 2023 Impact of Inflammatory Bowel Disease in Canada: Special Populations—Children and Adolescents with IBD

**DOI:** 10.1093/jcag/gwad016

**Published:** 2023-09-05

**Authors:** Wael El-Matary, Matthew W Carroll, Colette Deslandres, Anne M Griffiths, M Ellen Kuenzig, David R Mack, Eytan Wine, Jake Weinstein, Rose Geist, Tal Davis, Justin Chan, Rabia Khan, Priscilla Matthews, Gilaad G Kaplan, Joseph W Windsor, Charles N Bernstein, Alain Bitton, Stephanie Coward, Jennifer L Jones, Kate Lee, Sanjay K Murthy, Laura E Targownik, Juan-Nicolás Peña-Sánchez, Noelle Rohatinsky, Sara Ghandeharian, James H B Im, Quinn Goddard, Julia Gorospe, Jules Verdugo, Samantha A Morin, Taylor Morganstein, Lisa Banning, Eric I Benchimol

**Affiliations:** Department of Pediatrics and Child Health, Max Rady College of Medicine, University of Manitoba, Winnipeg, Manitoba, Canada; Division of Pediatric Gastroenterology and Nutrition, University of Alberta, Edmonton, Alberta, Canada; Department of Pediatrics, Division of Gastroenterology, Hepatology, and Nutrition, Centre Hospitalier Universitaire Sainte-Justine, Université de Montréal, Montréal, Québec, Canada; SickKids Inflammatory Bowel Disease Centre, Division of Gastroenterology, Hepatology, and Nutrition, The Hospital for Sick Children, Toronto, Ontario, Canada; Child Health Evaluative Sciences, SickKids Research Institute, The Hospital for Sick Children, Toronto, Ontario, Canada; Department of Paediatrics, Temerty Faculty of Medicine, University of Toronto, Toronto, Ontario, Canada; SickKids Inflammatory Bowel Disease Centre, Division of Gastroenterology, Hepatology, and Nutrition, The Hospital for Sick Children, Toronto, Ontario, Canada; Child Health Evaluative Sciences, SickKids Research Institute, The Hospital for Sick Children, Toronto, Ontario, Canada; CHEO IBD Centre and Department of Pediatrics, University of Ottawa, Ottawa, Canada; Departments of Pediatrics and Physiology, University of Alberta, Edmonton, Alberta, Canada; Edmonton Pediatric IBD Clinic, Edmonton, Alberta, Canada; SickKids Inflammatory Bowel Disease Centre, Division of Gastroenterology, Hepatology, and Nutrition, The Hospital for Sick Children, Toronto, Ontario, Canada; Child Health Evaluative Sciences, SickKids Research Institute, The Hospital for Sick Children, Toronto, Ontario, Canada; Department of Psychiatry, The Hospital for Sick Children, University of Toronto, Toronto, Canada; SickKids Inflammatory Bowel Disease Centre, Division of Gastroenterology, Hepatology, and Nutrition, The Hospital for Sick Children, Toronto, Ontario, Canada; Child Health Evaluative Sciences, SickKids Research Institute, The Hospital for Sick Children, Toronto, Ontario, Canada; Department of Pediatrics, Division of Gastroenterology, Hepatology, and Nutrition, British Columbia Children Hospital Research Institute, Vancouver, British Columbia, Canada; SickKids Inflammatory Bowel Disease Centre, Division of Gastroenterology, Hepatology, and Nutrition, The Hospital for Sick Children, Toronto, Ontario, Canada; Child Health Evaluative Sciences, SickKids Research Institute, The Hospital for Sick Children, Toronto, Ontario, Canada; ICES, Toronto, Ontario, Canada; Department of Medicine, McMaster University, Hamilton, Ontario, Canada; Departments of Medicine and Community Health Sciences, University of Calgary, Calgary, Alberta, Canada; Departments of Medicine and Community Health Sciences, University of Calgary, Calgary, Alberta, Canada; Department of Internal Medicine, Max Rady College of Medicine, Rady Faculty of Health Sciences, University of Manitoba, Winnipeg, Manitoba, Canada; University of Manitoba IBD Clinical and Research Centre, Winnipeg, Manitoba, Canada; Division of Gastroenterology and Hepatology, McGill University Health Centre, IBD Centre, McGill University, Montréal, Quebec, Canada; Departments of Medicine and Community Health Sciences, University of Calgary, Calgary, Alberta, Canada; Departments of Medicine, Clinical Health, and Epidemiology, Dalhousie University, Halifax, Nova Scotia, Canada; Crohn’s and Colitis Canada, Toronto, Ontario, Canada; Department of Medicine, University of Ottawa, Ottawa, Ontario, Canada; The Ottawa Hospital IBD Centre, Ottawa, Ontario, Canada; Division of Gastroenterology and Hepatology, Mount Sinai Hospital, Toronto, Ontario, Canada; Department of Community Health and Epidemiology, University of Saskatchewan, Saskatoon, Saskatchewan, Canada; College of Nursing, University of Saskatchewan, Saskatoon, Saskatchewan, Canada; Crohn’s and Colitis Canada, Toronto, Ontario, Canada; SickKids Inflammatory Bowel Disease Centre, Division of Gastroenterology, Hepatology, and Nutrition, The Hospital for Sick Children, Toronto, Ontario, Canada; Child Health Evaluative Sciences, SickKids Research Institute, The Hospital for Sick Children, Toronto, Ontario, Canada; Departments of Medicine and Community Health Sciences, University of Calgary, Calgary, Alberta, Canada; Departments of Medicine and Community Health Sciences, University of Calgary, Calgary, Alberta, Canada; Crohn’s and Colitis Canada, Toronto, Ontario, Canada; Department of Medical Sciences, McMaster University, Hamilton, Ontario, Canada; Faculty of Medicine and Health Sciences, McGill University, Montreal, Quebec, Canada; Crohn’s and Colitis Canada, Toronto, Ontario, Canada; SickKids Inflammatory Bowel Disease Centre, Division of Gastroenterology, Hepatology, and Nutrition, The Hospital for Sick Children, Toronto, Ontario, Canada; Child Health Evaluative Sciences, SickKids Research Institute, The Hospital for Sick Children, Toronto, Ontario, Canada; Department of Paediatrics, Temerty Faculty of Medicine, University of Toronto, Toronto, Ontario, Canada; ICES, Toronto, Ontario, Canada; Institute of Health Policy, Management and Evaluation, Dalla Lana School of Public Health, University of Toronto, Toronto, Ontario, Canada

**Keywords:** Crohn’s disease, Ulcerative colitis, Paediatrics, Transition

## Abstract

Rates of inflammatory bowel disease (IBD) in Canadian children and adolescents are among the highest in the world, and the incidence is rising most rapidly in children under five years of age. These young children may have either a typical form of IBD with multi-factorial aetiology, or they may have a monogenic form. Despite the growing number of children in Canada living with this important chronic disease, there are few available medical therapies approved by Health Canada due to the omission of children from most clinical trials of newly developed biologics. As a result, off-label use of medications is common, and physicians have learned to use existing therapies more effectively. In addition, most Canadian children are treated in multidisciplinary, specialty clinics by physicians with extra training or experience in IBD, as well as specialist nurses, dietitians, mental health care providers and other allied health professionals. This specialized clinic approach has facilitated cutting edge research, led by Canadian clinicians and scientists, to understand the causes of IBD, the optimal use of therapies, and the best ways to treat children from a biopsychosocial perspective. Canadians are engaged in work to understand the monogenic causes of IBD; the interaction between genes, the environment, and the microbiome; and how to address the mental health concerns and medical needs of adolescents and young adults transitioning from paediatric to adult care.

**Key Points**

1. The number of new diagnoses of children with IBD is rising rapidly in Canada, particularly in those with onset <6 years old (Very Early Onset IBD [VEO-IBD]).
2. Our understanding of the aetiology of childhood-onset IBD and the reasons for its rising incidence is still rudimentary. More research into the interaction between environmental factors, genetics, the gut microbiome, and the host immune system may allow us a better understanding of treatment and preventive opportunities.
3. IBD presenting in childhood is different from IBD presenting in adulthood. Children have more extensive disease, higher rates of acute severe colitis, and are at risk for linear growth delay, pubertal delay, and bone development deficits. In addition, children with VEO-IBD are more likely to present with isolated colonic disease. These differences have important implications on treatment choice, such as the avoidance of corticosteroids in favour of dietary therapy or biologics.
4. Children with IBD and their families have unique healthcare needs. These may result from differences in physical manifestations of IBD, as well as important differences in mental health (higher rates of anxiety and depression) and social well-being (stress on the child and family, missed school for the individual, missed work for caregivers).
5. The treatment options for children with IBD are limited, especially considering there are fewer choices for Health Canada approved medications and biologics. However, ongoing research aims to provide a better understanding of how to use available treatments more safely and effectively.
6. There is a growing need to understand how to optimize medical therapy in children to achieve better prognoses. A precision health approach to the treatment of IBD holds great promise for the future.
7. The period of transition from paediatric to adult care is one where adolescents and young adults with IBD may be at-risk for physical and psychosocial difficulties. More research is required to understand the risks to these individuals, and the best way to avoid them.
8. Approximately 3% of children with IBD have a monogenic form of the disease; this is more common in children who present with VEO-IBD (7.8%) than those diagnosed between 6 and 18 years of age (2.3%). Canadian researchers are leading international studies to understand the causes of this form of monogenic IBD, and new therapies may be developed to treat these children.
9. It is important for physicians and the public to recognize that IBD can occur in young children and access to specialist diagnosis and multidisciplinary care should be facilitated by the healthcare systems.

## SUMMARY OF CROHN’S AND COLITIS CANADA’S 2018 IMPACT OF INFLAMMATORY BOWEL DISEASE IN CANADA: SPECIAL POPULATIONS—CHILDREN WITH IBD

The incidence and prevalence of paediatric inflammatory bowel disease (IBD) in 2018 was high in Canada, and rates of Very Early Onset IBD (diagnosis at <6 years of age) were rising rapidly. Children with IBD present differently from adults and face unique health challenges such as growth failure, osteoporosis, more extensive disease, and difficulty adapting to a chronic disease during adolescence. The mental health and psychosocial well-being of children with IBD and their families are of utmost importance. Nevertheless, inadequate resources were provided for this important care. Treatments for paediatric IBD differ from adults, and there was a paucity of data from clinical trials to support the use of many medications frequently used to treat children. There are gaps in our knowledge of why paediatric IBD is rising in Canada; the best ways to provide medical, dietary, and psychosocial care to children and their families; and how to reduce variation in care to individuals with IBD.

## INTRODUCTION: CHILDREN AND ADOLESCENTS WITH INFLAMMATORY BOWEL DISEASE

Unlike adult IBD, the incidence of paediatric IBD continues to rise across the globe (1). In addition, rates of new diagnoses seem to be rising most rapidly in children under six years old, those classified as having Very Early Onset IBD (VEO-IBD) (1,2). Just as in adults, Canada has among the highest rates of paediatric IBD in the world. The incidence of childhood-onset IBD is 9.68 per 100,000 children, ranging from 7.22 per 100,000 in Manitoba to 15.18 per 100,000 in Nova Scotia (2). The prevalence of children <16 years of age living with IBD is 38.25 per 100,000 (2). While this is lower than the prevalence in adults, children face unique biologic and psychosocial needs when living with this chronic disease, and there are far fewer medical therapies approved for use in children by Health Canada. This situation makes the care of children with IBD, and the challenges faced by these children and their families, important healthcare concerns in Canada. This chapter reviews some important concepts in paediatric IBD to identify the needs for clinical care and research in this important population.

## ENVIRONMENTAL RISK FACTORS

The aetiopathogenesis of IBD is incompletely understood. The current hypothesis is that in genetically susceptible individuals, an environmental trigger (or triggers) will result in an inappropriate immune response. Disturbance of the normal gut microbiome (i.e., dysbiosis) may be associated with this dysregulated immune response. This response may be caused by the inflammation, may result from the environmental exposures, or both (3).

The association between many environmental factors and IBD has been investigated. In the paediatric age group, harmful factors include early exposure to antibiotics (4–6). Among the protective factors, breastfeeding was protective against Crohn’s disease (odds ratio [OR]: 0.71; 95% confidence interval [CI]: 0.59, 0.85) and ulcerative colitis (OR: 0.78; 95% CI: 0.67, 0.91) (7). Living conditions during childhood may affect disease development, with a generally protective role for rural residence (8). Residential greenspace during the childhood period was associated with a lower risk of developing paediatric-onset IBD (hazard ratio [HR]: 0.77; 95% CI: 0.74, 0.81) (9). Living near a farm with animals, bed sharing, and having pets during childhood had protective roles against IBD (10). Fruit intake protected against Crohn’s disease (OR: 0.57; 95% CI: 0.44, 0.74) and ulcerative colitis (OR: 0.69; 95% CI: 0.49, 0.96) (10). No evidence of association between carbohydrate, sugar, protein, or fat intake and either ulcerative colitis or Crohn’s disease was noted. However, studies in adults have demonstrated an association between high fat diets and IBD (11). In Manitoba, mode of delivery at birth did not seem to affect IBD development (12). The association between air pollution and the development of IBD is controversial (13). A UK study demonstrated an association in people with IBD onset <23 years old (14), while a Canadian study did not find an association between regional air pollution and the development of paediatric-onset IBD (15).

## DIFFERENCE BETWEEN PAEDIATRIC AND ADULT IBD

There are substantial differences in disease location, phenotype, and severity in paediatric-onset IBD compared to adult-onset IBD. Reduction in linear growth rate (16,17), a decrease in bone mineral deposition (18), and delay in pubertal development (17,19) are the consequences of pro-inflammatory cytokines released from the inflamed intestine (20). Such complications may be present at the time of diagnosis, particularly in children with Crohn’s disease. However, greater awareness of IBD in children will hopefully result in less diagnostic delay, which has been associated with better growth and development (21,22). Adequate treatment of the inflammation, particularly with biologics, is now usually effective to address these complications before they become permanent (23).

**Figure 1.**
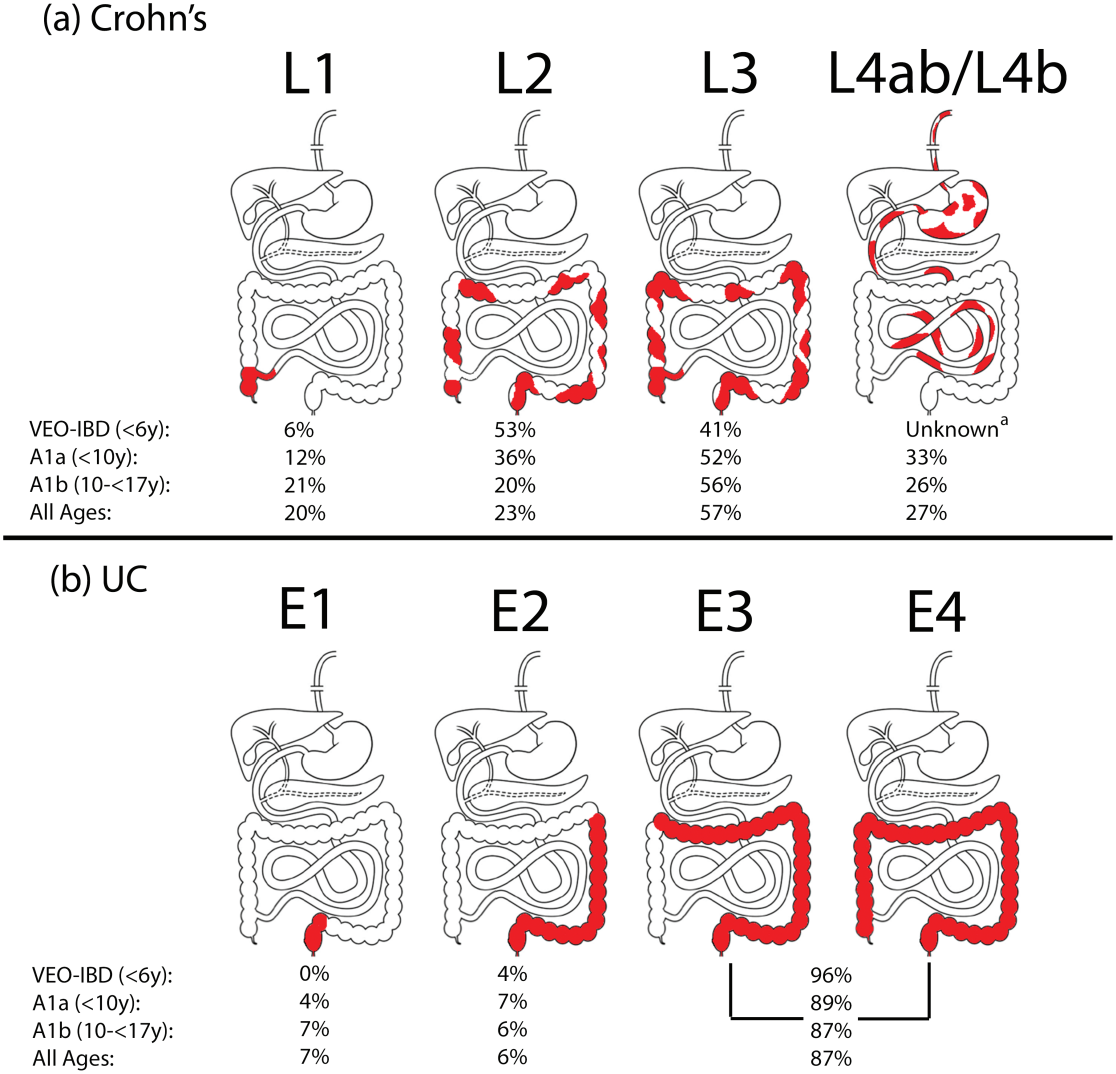
Location of childhood-onset (**a**) Crohn’s disease and (**b**) ulcerative colitis according to the Paris modification of the Montreal Classification (27). Adapted from Dhaliwal et al. (24) ^a^Frequency of L4ab/L4b phenotype was unknown in individuals with VEO-IBD because MR enterography was not performed in this age group.

Figure 1 demonstrates the frequency of disease phenotype for Crohn’s disease and ulcerative colitis derived from the inception cohort of the Canadian Children IBD Network (24). Overall, children typically have more extensive disease, which is more often considered severe; this is particularly well-documented for ulcerative colitis. Extensive or pancolitis predominate in paediatric-onset ulcerative colitis, whereas left sided disease is more common in adults (25). The most common disease phenotype in children with Crohn’s disease is inflammatory ileocolonic disease as compared to adulthood-onset Crohn’s disease with ileocecal predominance (25). Finally, there is a male predominance in paediatric-onset Crohn’s disease, which gradually becomes an equal ratio of male to female after puberty (26).

## TREATMENT OF PAEDIATRIC IBD

While medical therapies, including biologics and nutritional therapy, are highly effective in paediatric IBD, the treatment of children represents significant challenges. Only two biologics (infliximab and adalimumab) are currently approved by Health Canada for use in children, resulting in high rates of immunomodulator use and off-label use of other biologics (28). Children are typically excluded from initial clinical trials of biologics and other therapies, and paediatric trials only occur once the adult indication has been approved by regulators. Therefore, paediatric indications for medications are typically delayed by years, which has long been identified as a gap in the care of children with IBD (29,30). This problem is particularly pronounced for VEO-IBD, the most rapidly increasing incident group (2,28). A recent meeting of the Food and Drug Administration in the US determined ways in which clinical trials in children with IBD could be facilitated, hopefully improving the process in the future (31). Even when therapies are approved for use in children, dosage and monitoring do not follow adult guidelines, and data guiding the best use of these treatments are lacking (32). However, some improvements in our use of treatments in children with IBD are described below.

A gap in our use of medications to treat children with IBD is understanding the outcomes of greatest relevance to children and their families; these were previously not well-reflected in paediatric trials, which used disease activity scales as their primary outcomes. However, this situation is improving. The International Organization for the study of IBD (IOIBD) recently updated the STRIDE-II guidelines, which now also included paediatric-specific targets for outcomes (normalization of linear growth, paediatric-specific disease activity scores, and mucosal healing) (33). In addition, paediatric clinical trials are evolving to include objective measures such as mucosal healing, as well as patient-centred outcomes such as function, disability, and health-related quality of life (34). These initiatives will likely change the design and conduct of paediatric clinical trials in the future, making them more relevant to children with IBD and their families, as well as paediatricians.

In the meantime, we have learned to use existing paediatric therapies more effectively. Use of biologics earlier in the course of disease is increasingly common in Canada, including use of anti-TNF biologics as first-line therapy in children (35). A recent randomized clinical trial confirmed that this strategy is superior to conventional therapies (defined as corticosteroids and/or exclusive enteral nutrition) in achieving short- and long-term outcomes in children with Crohn’s disease, indicating the need to revise our treatment approach (36). A recent trial in children with Crohn’s disease using adalimumab found that proactively monitoring serum drug levels and adjusting the dose to meet the targeted range resulted in better outcomes after 72 weeks of therapy (37), indicating the need for an individualized approach to the treatment of each person. In addition, it is clear that some children require larger doses of biologics when weight-based dosing is used (32). Body surface area-based dosing has been demonstrated to be more effective in young children with IBD, and frequently results in exposure to higher doses of biologics and serum titres than weight-based dosing (38,39).

There has been renewed interest in various dietary therapies and their effects on gut inflammation and microbiome. The Crohn’s Disease Exclusion Diet (CDED) combined with partial enteral nutrition has been shown as effective as exclusive enteral nutrition for induction of remission of Crohn’s disease and was better tolerated by children (40). The CDED is based on the removal of various elements of the Western diet thought to promote gut inflammation, such as animal fats, processed foods, dairy, and wheat and has been associated with partial correction of dysbiosis (41). It was also demonstrated to be effective for inducing and maintaining remission in a pilot trial of adults with Crohn’s disease (42). Dietary therapies and other methods of manipulating the gut microbiome to induce remission and aid with healing are currently being trialled in Canadian centres (43). Finally, fecal microbial transplantation is the subject of a Canadian clinical trial in children with ulcerative colitis (44). For further information on the role of diet and nutrition in the treatment of paediatric IBD, and on fecal microbial transplant, see Murthy et al. (this volume).

Another key theme of paediatric IBD care in the past five years has been predicting which children are at increased risk for negative outcomes. Multiple studies have identified risk factors for hospitalizations, surgeries, and complications from Crohn’s disease (45–48), as well as hospitalization or colectomy in ulcerative colitis (49). For example, a recent retrospective Canadian study found that female sex, 5ASA medication use, immunomodulator use (instead of anti-TNF biologics), granulomas and eosinophils on biopsies, elevated inflammatory markers during clinical remission, and lower serum infliximab titres during clinical remission were associated with clinical relapse (50). Two recent systematic reviews from the Pediatric IBD-Ahead group summarized the literature on risk prediction for children with Crohn’s disease and ulcerative colitis (51,52). While this body of literature is useful to identify those at increased risk, observational research cannot prove that treating earlier or more aggressively will avoid long-term failures of medical therapy. In addition, most of these risk factors have been difficult to validate in external, prospective cohort studies (53). In light of these barriers, research into a precision health approach to IBD care has become a priority. Precision health is defined as the integration of genetics, proteomic, metabolomic, microbiome, environmental, and sociodemographic characteristics to create a personalized approach to the care of each individual living with IBD (54).

Multidisciplinary care (involving physicians, nurses, dieticians, and mental health care providers) has been identified as important for optimization of outcomes in children and adolescents with IBD (55,56). Studies have identified mental illness as a significant predictor of nonadherence to medications (57), and of high direct healthcare costs in children (58). Nevertheless, access to multidisciplinary care varies widely amongst paediatric IBD care centres in Canada, with access to mental health care being widely considered inadequate, even in large paediatric hospitals (59).

In summary, regulatory approval of medications for the treatment of paediatric IBD lags behind approval in adults, and a regulatory framework for drug approval and labelling in paediatrics is overdue (60). In addition, drug cost reimbursement (whether by private or public payors) should change to reflect the latest scientific evidence rather than relying on old research and the clinical trials that resulted in regulatory approval. Nevertheless, Canadian paediatric IBD care providers have spearheaded new and unique ways of using existing therapies, more effectively making use of anti-TNF biologics, while learning more about the off-label usage of newer medications and dietary therapies. As such, the outcomes of children with IBD have improved markedly over the past 20 years, with reduced rates of hospitalization and surgery (61). However, the provision of multidisciplinary care may result in better clinical outcomes, quality of life, and direct healthcare costs in children with IBD.

## IMPACT ON INDIVIDUALS, PARENTS, AND CAREGIVERS

The impact of any paediatric chronic disease—especially IBD—on children and young adults, their parents, and families can be substantial. Although incurable, thanks to newer biologics, treatment approaches, and specialized care, most children with IBD can largely remain well and be expected to live a full and happy life. However, coping with the diagnosis of IBD, and its associated active and recurrent symptoms, treatment costs, and potential adverse events can be challenging. Children spend the majority of their time within a larger family system, and family functioning is bound to influence a child’s adjustment to IBD (62). Considering physical and psychological burdens of IBD, it is not surprising that these individuals are at risk of struggling and coping from a mental health perspective (63). Mental health concerns of children with IBD is addressed in detail in Graff et al. (this volume).

Parents play an important part in managing their children’s IBD. They help with explaining the disease to their children, medication adherence, scheduling different medical appointments, and establishing a successful relationship with physicians. Balancing these roles may increase the stress on parents and caregivers. Hence, parents of children with IBD were found to experience greater emotional distress, depression, and lack of emotional support, as compared to parents of healthy children (63). There is a positive correlation between parental stress and internalization of symptoms (depression, anxiety, somatization) among children with IBD (63). More parental stress was associated with more severe disease and lower health-related quality of life among their children with IBD (63). Conversely, more parental involvement was associated with higher rates of adherence to treatments for IBD (64).

## EDUCATION AND FUTURE EMPLOYMENT

Missing school days due to IBD-related causes, such as attending clinics and hospitalizations was common in children with IBD, especially in those with active disease (65). Parental satisfaction with education of children with IBD attending advanced secondary education was also found to be lower in a cross-sectional study, especially in individuals with active disease (66). However, despite these challenges, Manitoba children with IBD had school performance equal to those of other children as long as they did not struggle with mental illness (67).

Data on employment and income potential in people with paediatric-onset IBD is heterogeneous. A recent Swedish population-based study found that people with childhood-onset IBD reported lower income between ages of 20–30 compared to healthy controls, especially in those who needed prolonged periods of hospitalizations or surgery (68). Conversely, a Canadian study reported higher future long-term adulthood earnings in people with paediatric-onset IBD compared to healthy controls (69). The difference between the two studies may be related to the difference in design and income data sources; administrative data were used in the Scandinavian study whereas a cross-sectional survey was administered in the Canadian study. The Canadian study had a longer duration of follow up, but smaller sample size. Both studies, however, showed no difference in future unemployment and marital status in those with paediatric-onset IBD as compared to the general population (68,69).

## TRANSITION FROM PAEDIATRIC TO ADULT CARE

Transition in care is defined as the purposeful and planned movement of adolescents and young adults (AYAs) with a chronic medical condition to adult-oriented healthcare systems/care providers (70,71). Children in Canada transition from paediatric to adult healthcare services between the ages of 14–18, with ultimate transfer to adult care around the time the child turns 18 years. There are inherent differences between paediatric- and adult-care models; paediatric care is family focused, multidisciplinary, and has caregiver involvement for consent and guidance, while adult care typically has a single provider and adult providers expect that the individual will be capable of making decisions independently from their parents or caregivers (72,73). One stressor faced by children and adolescents with IBD (and other chronic diseases) is the everyday developmental transitions from childhood to adulthood, including changes in school structure, employment, general psychosocial growth, and changes in insurance coverage from the parents’ plan to the individual’s own coverage (74,75). These developmental transitions are particularly important for children with a chronic disease as they are amplified by the transfer from paediatric to adult healthcare systems.

There is no standard of care for transitioning adolescents with IBD in Canada and success of transition is defined differently by individuals, parents, and healthcare providers (76). However, Crohn’s and Colitis Canada recently partnered with the Canadian IBD Transition Network to produce expert consensus statements on best-practices for transitioning AYAs with IBD (77). Transition for AYAs with special healthcare needs has been identified as a health services priority area (78). Studies have demonstrated a higher economic burden among young adults with paediatric-onset IBD, including increased all-cause total healthcare costs and the highest utilization of emergency services of any sub-population (79,80). In a healthcare era plagued by economic constraints, ensuring positive healthcare outcomes via the most cost-efficient healthcare delivery is a priority. In Ontario, adolescents with IBD had more visits to the emergency department (ED) after transfer to adult gastroenterology care (81). However, these ED visits were not associated with an increased risk of hospitalization, suggesting that they were not due to a severe flare of IBD, and might have been avoided with adequate access to outpatient care and education regarding appropriate ED use (81,82). In Canada, care of children and adolescents with Crohn’s disease is almost exclusively provided in paediatric IBD centres affiliated with academic paediatric hospitals (59). However, in a recent multi-centre Canadian study of adolescents aged 16–19 years, only 26.6% of adolescents treated in paediatric centres met criteria for readiness to be transferred to adult care (83). In addition, these individuals had a significant burden of mental health concerns (83).

Crohn’s and Colitis Canada has partnered with the Leona M. and Harry B. Helmsley Charitable Trust to evaluate an intervention to smooth the transition from paediatric to adult care (84). This randomized controlled trial of a biopsychosocial and educational intervention stands to provide the highest level of evidence of an intervention to improve transition for individuals with IBD.

## VEO-IBD AND MONOGENIC IBD

VEO-IBD is defined relatively arbitrarily as IBD diagnosed before the age of six years (85). The highest percentage increases of incidence in Canada have been observed in this age group (2). The majority of these young children have complex IBD, that is Crohn’s disease or ulcerative colitis developing due to genetic and environmental factors (86). However, over the last two decades, advances in genomic analyses have discovered multiple monogenic causes of chronic IBD-like diseases. These are immune disorders secondary to genetic mutations and are associated with severe inflammation of the gastrointestinal tract, often not responsive to conventional IBD therapy. In addition, these individuals commonly have multiple system involvement (87). A recent Canadian study that included over 1,000 children with IBD reported results of whole exome sequencing of the 68 genes known to cause monogenic IBD. They reported that 3.4% of the cohort overall, but 13.8% of children younger than two years at diagnosis (infantile-onset IBD), and 7.8% of children with VEO-IBD were found to have a disease-causing mutation in one of these genes (86). Hematopoietic stem cell transplantation (HSCT) can cure several of these disorders such as X-linked inhibitor of apoptosis protein deficiency and chronic granulomatous diseases as it can correct the immune defects in these orders (88). Unfortunately, HSCT is ineffective for epithelial barrier dysfunctions such as nuclear factor-kappa B essential modulator deficiency as it cannot change the expression of these proteins on the intestinal epithelium (88). Some of these disorders, however, may respond to specific medications; for example, mevalonate kinase deficiency may respond to IL-1 receptor antagonists (89). Canadian researchers are leading an international consortium to find new genetic causes and treatments for VEO-IBD (NEOPICS: the InterNational Early Onset Paediatric IBD Cohort Study).

## KNOWLEDGE GAPS AND FUTURE RESEARCH DIRECTIONS

- Understanding the causes of childhood-onset IBD and the reasons for its increased incidence in Canada and worldwide will help us identify potential treatments and preventive strategies to improve outcomes and reduce the risk.
- Much more research is required to understand the mental health, psychosocial, educational, and employment implications of having IBD on children and their families, and therefore the resources required to improve their quality of life.
- Better interventions are required to improve the transition from pediatric to adult care for adolescents and young adults with IBD.
- Children with IBD have access to a limited number of approved therapies. We must learn to use these therapies more effectively and efficiently.
- A precision medicine approach to the treatment of IBD may help improve outcomes of all children with IBD, including those with monogenic forms.

## PATIENT AND CAREGIVER PERSPECTIVE

Patient partners recognized that even though the cases of IBD are rising in children, there are limited medication options available in this age group as they are often not included in clinical trials. It gave them hope to learn more about single gene mutations that may cause a subset of IBD because new therapies may be developed to directly treat this form of IBD. It was identified by patient partners that there was no standardized transition process from paediatric to adult care across Canadian provinces. Patient partners suggested that greater emphasis should be placed on implementing consistent and individualized transition plans across the country. The identification that greater research was needed in the areas of causes and risk factors towards the development of IBD as well as medical treatments, mental health, and psychosocial implications to improve patient and family experience and quality of life provides patient partners with feelings of recognition and hope for the future.

## POLICY IMPLICATIONS AND KEY ADVOCACY OUTCOMES

1. Rates of paediatric IBD are rising in Canada. These individuals require multi-disciplinary and specialized care for their chronic disease. They should have access to expert physicians, nurses, dieticians, social workers, pharmacists, and mental health specialists to treat both the individual and their family, no matter where in Canada they live.
2. The development of IBD in childhood has lifelong implications for the individual and family, and we need to better educate healthcare providers, policy-makers, and the general public about the challenges faced by those with childhood-onset IBD.
3. Crohn’s and Colitis Canada should advocate for the inclusion of children and adolescents in industry-sponsored and investigator-initiated clinical trials to allow for a better understanding of their efficacy in children and adolescents, and for earlier regulatory approval of these new medications.
4. Advocacy efforts should also focus on improved funding of research to understand how to better use available treatments in children with IBD to improve outcomes.
5. Specific attention should be given to creating an evidence-based standard of care for those transitioning from paediatric to adult care.
6. Education/awareness should be provided to the public, afflicted individuals, and healthcare providers, especially primary care providers, so that children with IBD can be identified, referred to a specialist, and diagnosed quickly, and appropriate care pathways followed.

## Data Availability

No new data were generated or analyzed in support of this review.
